# The Role of PPARγ Ligands in Breast Cancer: From Basic Research to Clinical Studies

**DOI:** 10.3390/cancers12092623

**Published:** 2020-09-14

**Authors:** Giuseppina Augimeri, Cinzia Giordano, Luca Gelsomino, Pierluigi Plastina, Ines Barone, Stefania Catalano, Sebastiano Andò, Daniela Bonofiglio

**Affiliations:** 1 Department of Pharmacy, Health and Nutritional Sciences, University of Calabria, 87036 Arcavacata di Rende (CS), Italy; giusy.augimeri@gmail.com (G.A.); cinzia.giordano@unical.it (C.G.); luca.gelsomino@unical.it (L.G.); pierluigi.plastina@unical.it (P.P.); ines.barone@unical.it (I.B.); stefcatalano@libero.it (S.C.); sebastiano.ando@unical.it (S.A.); 2 Centro Sanitario, University of Calabria, 87036 Arcavacata di Rende (CS), Italy

**Keywords:** peroxisome proliferator-activated receptor gamma, synthetic *PPAR*γ agonists, thiazolidinediones, selective PPARγ modulators, nonsteroidal anti-inflammatory drugs, natural *PPAR*γ agonists, 15-deoxy-D12,14-prostaglandin J2, omega-3 polyunsaturated fatty acids, PUFA

## Abstract

**Simple Summary:**

Breast cancer represents the most frequently diagnosed carcinoma and the leading cause of cancer death in women. Despite advances achieved in systemic therapy, about one-third of all patients relapse and develop a metastatic disease, which ultimately leads to breast cancer deaths. In this scenario, the identification of new prognostic factors and pharmacological tools is needed to improve breast cancer management. Peroxisome proliferator-activated receptor gamma (PPARγ), belonging to the nuclear receptor superfamily, is a ligand-dependent transcription factor expressed in many tumors including breast cancer, and its function upon binding of ligands has been linked to the tumor development, progression and metastasis. Over the last decade, much research has focused on the implication of natural and synthetic PPARγ agonists in the negative regulation of breast cancer growth and progression. The aim of the present review is to summarize the role of PPARγ activation in breast cancer from the basic research to clinical studies. The therapeutic effects of natural and synthetic PPARγ ligands, as antineoplastic agents, represent a fascinating and clinically a potential translatable area of research with regards to the battle against cancer.

**Abstract:**

Peroxisome proliferator-activated receptor gamma (PPARγ), belonging to the nuclear receptor superfamily, is a ligand-dependent transcription factor involved in a variety of pathophysiological conditions such as inflammation, metabolic disorders, cardiovascular disease, and cancers. In this latter context, PPARγ is expressed in many tumors including breast cancer, and its function upon binding of ligands has been linked to the tumor development, progression, and metastasis. Over the last decade, much research has focused on the potential of natural agonists for PPARγ including fatty acids and prostanoids that act as weak ligands compared to the strong and synthetic PPARγ agonists such as thiazolidinedione drugs. Both natural and synthetic compounds have been implicated in the negative regulation of breast cancer growth and progression. The aim of the present review is to summarize the role of PPARγ activation in breast cancer focusing on the underlying cellular and molecular mechanisms involved in the regulation of cell proliferation, cell cycle, and cell death, in the modulation of motility and invasion as well as in the cross-talk with other different signaling pathways. Besides, we also provide an overview of the in vivo breast cancer models and clinical studies. The therapeutic effects of natural and synthetic PPARγ ligands, as antineoplastic agents, represent a fascinating and clinically a potential translatable area of research with regards to the battle against cancer.

## 1. Introduction

Breast cancer represents the most frequently diagnosed carcinoma and the leading cause of cancer death in women [1]. In 2018, 2.1 million new cases and 626,679 deaths were recorded, making the management of breast cancer a public health priority [2]. Based on the gene expression of the standard molecular markers, estrogen receptor α (ERα), progesterone receptor (PR), and human epidermal growth factor receptor 2 (HER2), breast cancer is categorized in four main subtypes: Luminal A (ERα and/or PR positive and HER2 negative); Luminal B (ERα and/or PR positive and HER2 positive or negative); HER2-positive (ERα/PR negative and HER2 positive); Triple-negative, TNBC (ERα/PR/HER2 negative), each characterized by different prognosis and response to drug treatment [3,4]. Currently, locoregional (surgery and radiotherapy) and systemic (chemotherapy, endocrine, and biological therapy) approaches represent the main therapeutic options for the treatment of breast cancer [5]. Despite advances achieved in systemic therapy, approximately 30% of patients relapse and develop a metastatic disease, which ultimately leads to breast cancer deaths [6,7]. Particularly, patients with TNBC, which accounts for 10–20% of all cases of breast carcinoma, have a relatively poor outcome and require additional treatment approaches since this tumor subtype is characterized by the lack of biomarker expression [8]. In this scenario, the identification of new prognostic factors and pharmacological tools is needed to improve breast cancer management. Apart from ERα and PR, numerous nuclear receptors, including the androgen receptor (AR), the vitamin D3 receptor (VDR), the retinoid X receptors (RXR), and the farnesoid X receptor (FXR) have been investigated as potential novel targets for the treatment of breast cancer because of their role as key regulators of gene expression [9,10,11,12]. In the last decades, the peroxisome proliferator-activated receptor γ (PPARγ), a ligand-activated transcription factor, which has been mainly characterized as a pivotal regulator of adipocyte differentiation, has also received considerable attention for its role in breast cancer tumorigenesis [13,14]. Clinical studies revealed that PPARγ expression represents a positive prognostic factor in luminal and ductal breast cancer patients [15], since higher levels of PPARγ inversely correlate with grade, tumor size, and TNM staging system of malignant tumor [16,17]. Interestingly, activation of PPARγ by its natural and synthetic agonists has been found to modulate the expression of several genes associated with tumorigenesis, further highlighting that this nuclear receptor could represent a new promising target for the treatment of breast cancer. Synthetic PPARγ agonists, including the antidiabetic drugs thiazolidinediones (TZDs), demonstrated to induce growth inhibition, apoptosis, and differentiation of breast cancer cells in in vitro and in vivo models [18]. Although clinical trials in breast cancer patients fail to show the expected therapeutic values of TDZs [19], encouraging results have been obtained using natural PPARγ ligands in breast cancer. These compounds, including omega-3 (ω-3) fatty acids and their derivatives, have proven to be promising therapeutic agents in the prevention and treatment of breast cancer [20,21,22]. We herein summarize the current knowledge of the role of PPARγ and its natural and synthetic ligands in breast cancer providing an overview from in vitro experiments to in vivo and clinical studies. This review could allow a better comprehension of the PPARγ agonists as potential pharmacological or nutritional compounds against breast cancer.

## 2. Search Strategy and Data Extraction

A search for original articles published between 1997 and 2020 was performed in PubMed. The literature search was performed using the following keywords: “PPARγ” AND “cancer” obtaining 3774 papers. A total of 647 published papers were restricted when we used as keywords “PPARγ” AND “breast cancer.” In order to focus our attention on PPARγ ligands, “natural PPARγ ligands” AND “breast cancer” (37 papers) or “synthetic PPARγ ligands” AND “breast cancer” (46 papers) or “ω 3” AND “PPARγ” AND “breast cancer” (22 papers) keywords were used. All articles selected were full-text articles written in English. After removing the duplicates, the articles were screened based on their relevance to the topic and all irrelevant papers were excluded. We also identified further relevant articles from the reference lists of selected papers. The data reviewed have been organized in separate sections including: (1) structure, distribution, and mechanism of action of PPARγ; (2) synthetic and natural PPARγ ligands; and (3) role of the PPARγ ligands in breast cancer. Finally, PPARγ ligands have been highlighted as potential breast cancer therapeutic agents both from a nutritional and a pharmacological perspective.

## 3. Peroxisome Proliferator-Activated Receptor γ: Structure, Distribution, and Mechanism of Action

### 3.1. Genomic Organization and Tissue Distribution of PPARγ

PPARγ is a ligand-activated transcription factor belonging to the PPARs family, which also comprises PPARα and PPARβ/δ [23]. The human PPARγ gene, *NR1C3*, is located on the chromosome 3p25.2 and contains about 9 exons, which give rise to three different PPARγ transcripts (γ1, γ2, and γ3) by alternative promoter usage and splicing [24,25]. PPARγ1 and PPARγ3 mRNAs encode the same protein, whereas PPARγ2 protein contains 30 additional amino-acids in the N-terminal region. PPARγ1 mRNA has ubiquitous expression, although the highest levels were found in the adipose tissue; PPARγ2 mRNA is mainly expressed in the adipocytes; PPARγ3 mRNA has been highly found in macrophages and adipose tissue [26]. However, regardless of PPARγ subtypes, several types of cells express different levels of PPARγ. High levels of PPARγ protein expression were found in the adipose tissue, large intestine, and hematopoietic cells, whereas lower amounts of PPARγ were revealed in the kidney, liver, and small intestine [24]. Interestingly, PPARγ is also expressed in different types of tumors, including colon, prostate, and breast cancer [27]. PPARγ has been found not only in the epithelial cancer cells but also in several components of the tumor microenvironment [28,29,30], such as tumor-associated macrophages (TAMs) and cancer-associated fibroblasts (CAFs), suggesting that PPARγ may be a good target for integrative therapies affecting simultaneously tumors and their microenvironment.

### 3.2. PPARγ Domain Structure

**Figure 1:**
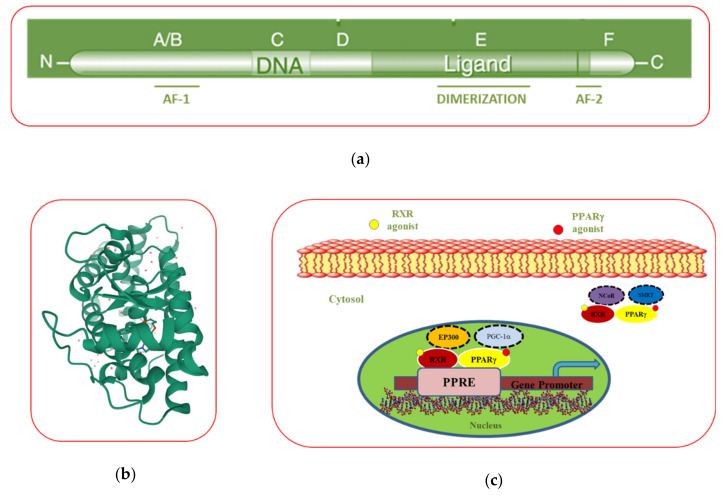
Modular domain (**a**) and crystal (**b**) structures of the peroxisome proliferator-activated receptor gamma (PPARγ). Ligand-binding activity of the PPARγ and retinoid X receptors (RXR) heterodimer, which is bound in the cytosol to the nuclear receptor corepressor complexes (N-CoR) or silencing mediator of retinoic acid and thyroid hormone receptor (SMRT), while in the nucleus is activated by PPARγ coactivator 1-α (PGC-1α) or binding protein p300 (EP300) for binding to the PPRE in the promoter of target genes (**c**).

The structural organization of PPARγ consists of five different domains named “A–E” from N- to C-terminus (Figure 1a). The N-terminus domain (A/B domain) is the most variable domain among the nuclear receptor family, and it contains the ligand-independent activation factor 1 (AF-1) region, that once phosphorylated modulates PPARγ activity [31]. The central DNA-binding domain (C domain) is the most conservative region, and it is involved in the binding of PPARγ to the DNA, after its heterodimerization with retinoid X receptor (RXR). The C domain consists of two zinc fingers composed of nine cysteines that recognize and bind the PPAR response elements (PPRE) in the promoter region of PPAR-response genes, regulating their transcription [31,32]. The consensus sequence of PPRE is composed of two direct repeat (DR)-1 elements consisting of two AGGTCA sequences separated by a single nucleotide spacer [31,33]. The D domain is a flexible hinge region connecting the C domain to the ligand-binding domain (E domain), which, in turn, is involved in the ligand recognition and binding [32]. Information from crystal structure has revealed that the ligand binding “pocket” of PPARγ is larger than other nuclear receptor, enabling the interaction of several compounds [23] (Figure 1b, https://www.rcsb.org/3d-view/2F4B/1). In the C-terminus, the F domain contains the ligand-dependent activation domain (AF-2), an amphipathic helix involved in the docking of coactivator proteins in response to ligand stimulation [31,34].

### 3.3. The Mechanism of Action of PPARγ

Like other members of the nuclear receptor family, PPARγ activity depends on its intracellular localization. In absence of ligands, PPARγ is localized in the cytoplasm bound to transcriptional corepressor complexes containing the nuclear receptor corepressor complexes (N-CoR) or silencing mediator of retinoic acid and thyroid hormone receptor (SMRT), which prevents PPARγ activation. Upon stimulation by own ligands, PPARγ undergoes a conformational change in AF2 domain which, through the replacement of the corepressor with the coactivator complex, consisting of proteins such as PPARγ coactivator 1-α (PGC-1α) or binding protein p300 (EP300), vitamin D receptor-interacting protein (DRIP), or thyroid hormone receptor-associated protein (TRAP), induces PPARγ activation [35,36]. Thus, PPARγ forms a heterodimer with RXR and acquires the ability to translocate into the nucleus and binds to the PPRE in the PPARγ target genes. Then, the coactivator multiprotein complex, containing histone acetylase enzymes, remodels the chromatin structure and allows the binding of RNA polymerase to the promoter of PPARγ target genes, initiating their transcription (Figure 1c) [37]. Till date, more than 100 genes have been identified as PPARγ-regulated factors, including those involved in lipid metabolism, glucose homeostasis, adipocyte differentiation, and tumorigenesis [38]. However, only a restricted number of functional PPREs have been discovered, limiting the possibility to completely categorized the PPARγ direct target genes. Indeed, the majority of the endogenous PPREs deviates from the consensus sequence and contains degenerate PPRE-like sequences, such as C-X-C chemokine receptor type 4 (CXCR4, AGGATAcAGATGA) [39], scavenger receptor class B member 1 (SR-BI, AGGAGAAAGG GGA) [40], and sodium-hydrogen antiporter 1 (NHE-1, GGTCAnnAGTTCG) [41] that are difficult to be identified since the extent of tolerable sequence variability in the PPRE is not completely understood [42]. Interestingly, it has been demonstrated that PPARγ ligands can cooperate with RXR ligands to modulate the transcription of PPARγ target genes [43]. In this view, PPARγ and RXR agonists may be used in low doses to achieve maximum pharmacological outcomes with low potential side effects [44,45]. PPARγ can regulate gene expression also through nongenomic mechanisms [31]. It has been demonstrated that PPARγ physically interacts with different transcription factors such as nuclear factor kappa-light-chain-enhancer of activated B (NFkB) and activator protein 1 (AP-1), blocking the expression of pro-inflammatory genes through transrepression mechanisms in a DNA-independent manner [46]. The anti-inflammatory effects of PPARγ have been extensively studied, but the mechanisms whereby PPARγ reduces the expression of pro-inflammatory genes are not completely understood [47]. Although the functions of PPARγ are mainly regulated through ligand binding, post-transcriptional modification, including phosphorylation, SUMOylation, ubiquitination, and nitration occur in specific sites of the AF-1 domain, regulating PPARγ activity [48]. The PPARγ domain and crystal structures along with the mechanism of action of this nuclear receptor are depicted in Figure 1.

## 4. PPARγ Agonists

### 4.1. Natural PPARγ Ligands

Over the last years, natural PPARγ ligands have received much attention because they activate PPARγ inducing numerous beneficial effects with fewer side effects compared to synthetic agonists. Natural PPARγ ligands include unsaturated fatty acids and their derivatives. In particular, PPARγ is preferentially activated by polyunsaturated fatty acids (PUFAs), such as ω-3 and ω-6 PUFAs derived from dietary sources [23,49]. ω-3 PUFAs, including eicosapentaenoic acid (EPA) and docosahexaenoic acid (DHA), and their derivatives, represent the most interesting class of natural PPARγ agonists since their dietary intake is associated with health benefits [50]. Indeed, several studies have reported that the consumption of ω-3-PUFA-rich foods, such as fish and walnuts, is associated with a decreased incidence of cardiovascular diseases and a reduced risk to develop different types of malignancy, including colorectal, prostate, and breast cancer [20,51,52]. In addition, it has been reported that consumption of ω-3 PUFAs correlates with increased insulin sensitivity [53] as well as improvement of the immune profile and cognition [54,55,56]. Among different molecular mechanisms by which ω-3 PUFAs exert their effects, it has been demonstrated that several ω-3 PUFA biological activities depend on the activation of PPARγ. Recently, it has been shown that DHA-rich fish oil supplementation reduces the triglyceride level in patients with type 2 diabetes in a PPARγ-dependent manner [57]. Interestingly, ω-3 PUFAs can be metabolized in intermediates, such as 17-hydroxy- and 7-hydroxy-DHA, which activate PPARγ more than parental compounds [58]. Moreover, ω-3 PUFAs conjugated with ethanolamine, dopamine, serotonin, or other amines have been extensively studied for their ability to activate PPARγ and are found to exert stronger effects than the unconjugated related compounds, but their molecular mechanism is not fully understood [59]. Recently, it has been demonstrated that the conjugates of DHA with ethanolamine (DHEA) or serotonin (DHA-5-HT) decrease the cytokine secretion by TAMs of breast cancer in a PPARγ-dependent manner, reprogramming TAMs in a less aggressive phenotype [30]. Apart from ω-3 PUFAs, ω-6 PUFAs and their derivatives are also natural PPARγ ligands. Moreover, 9-Hydroxyoctadecadienoic acid (9-HODE) and 13-Hydroxyoctadecadienoic acid (13-HODE) derived from ω-6 linoleic acid and 15-deoxy-Δ^12,14^-prostaglandin J2 (15d-PGJ2) obtained from ω-6 arachidonic acid are important endogenous PPARγ ligands, showing a broad range of effects, including anti-inflammatory and antineoplastic actions. Other molecules, such as nitrated fatty acids derived from nonenzymatic reactions of unsaturated fatty acids and endogenous nitric oxide, have been described as endogenous PPARγ ligands. These compounds, through PPARγ activation, induce anti-inflammatory effects, adipocyte differentiation and glucose uptake in in vitro models, but their biological role in humans is not clarified [60,61].

Other than PUFAs and their derivatives, numerous classes of bioactive compounds obtained from plant extracts, including flavonoids, neolignans, and sequiterpenes, have been identified as PPARγ ligands [62,63]. Most of them, such as genistein, quercetin, and luteolin displayed partial agonism profile on PPARγ. However, a complete categorization of bioactive compounds as PPARγ agonists is still missing because of the high number of active extracts that can induce PPARγ activation [62]. In Table 1, we have reported the most characterized natural PPARγ ligands.

### 4.2. Synthetic PPARγ Ligands

Although several synthetic molecules have shown affinity for PPARγ, TDZs represent the most important synthetic PPARγ ligands. These compounds, working as insulin sensitizers, are oral hypoglycemic agents suggested as second-line medication in the treatment of type 2 diabetes mellitus [76]. Troglitazone was the first TZD approved for clinical use, but it has been removed from the market in 2000 because of its liver toxicity. So far, Ciglitazone was never used as a medication, while rosiglitazone and pioglitazone are authorized for the treatment of type II diabetes as monotherapy or in combination with insulin, metformin or sulfonylurea, but their prescription and use are under several restrictions because of their potential side effects. Particularly, rosiglitazone was withdrawn from the market in Europe and in other several countries in 2010 because the benefits no longer outweighed the risks [77]. Edaglitazone is a potent and selective PPARγ agonist [78]. Recently, other two TDZ drugs, rivoglitazone and efatutazone, have been developed and are currently undergoing clinical trials [79,80]. The effects of TDZs depend on their ability to bind PPARγ and modulate PPARγ target gene expression through transactivation or transrepression mechanisms. Although the primary target tissue of TDZs is the adipose tissue, where PPARγ is most abundantly expressed, the hypoglycemic effects of TDZs depend on the insulin-stimulated glucose disposal in the skeletal muscle. However, the mechanism by which TDZs promote insulin action in nonadipocyte tissue is still unknown [35,81,82]. Despite TDZs are well tolerated, their administration may be responsible for a range of side effects, including edema, anemia, weight gain, and increased incidence of cardiovascular diseases [83]. In order to reduce the side effects of TDZ, partial PPARγ agonists, named “selective PPARγ modulators” (SPPARMs), have been developed, but they are not currently used in clinical practice [35,84,85]. In addition to TDZs, nonsteroidal anti-inflammatory drugs (NSAIDs) have been described as PPARγ agonists [86]. Recently, it has been demonstrated that NSAIDs, including sulindac sulfide, diclofenac, indomethacin, and ibuprofen, show different affinity to PPARγ and modulate its activity at pharmacological concentration, but the research in this direction is largely missing [87]. The most common synthetic PPARγ ligands are reported in Table 2.

## 5. The Role of the PPARγ Ligands in Breast Cancer

Since different cell lines, including breast, prostate, colon, bladder, and thyroid cancer cells express high levels of PPARγ, the potential role of PPARγ agonists in modulating cancer progression has been widely investigated [96,97]. A vast scientific literature about the effects of PPARγ agonists in breast cancer is currently available, reflecting the interest in the use of PPARγ ligands in breast cancer management. In the following sections, the recent knowledge regarding the effects of PPARγ agonists in breast cancer biology from in vitro experiments to in vivo and clinical studies will be discussed.

### 5.1. In Vitro Studies

Many investigations undertaken with the aim of assessing a direct effect of PPARγ agonists on different processes involved in breast carcinogenesis demonstrated that these molecules induce cell growth inhibition, trigger cell death, inhibit breast cancer cell motility, and invasion. Moreover, activated PPARγ may cross-talk with other signal transduction pathways antagonizing breast tumor proliferation.

#### 5.1.1. Regulation of Cell Growth and Cell Cycle

**Figure 2:**
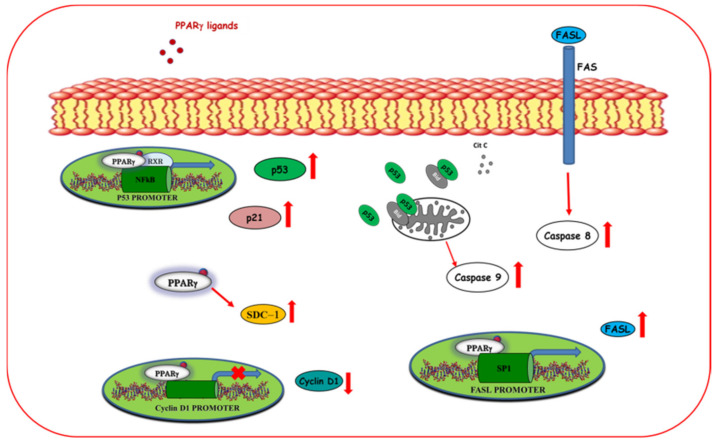
Molecular mechanisms by which ligand-activated PPARγ influences cell cycle and exerts proapoptotic effects in breast cancer cells. PPARγ ligands trans repressing the cyclin D1 promoter activity reduce its expression thereby inducing cell cycle arrest. Moreover, ligand activated PPARγ transactivates promoter gene p53 and enhances p53 protein expression and its target gene p21 triggering the intrinsic apoptotic pathways. In addition, PPARγ ligands upregulate the expression of syndecan-1 (SDC-1), inducing apoptosis. PPARγ ligands also trigger extrinsic apoptosis through activation of FAS ligand (FASL) gene promoter activity.

Uncontrolled cell proliferation is a hallmark of cancer cells based on the deregulated activity of cell cycle proteins [98,99]. Cyclin-dependent kinases (CDKs) and their regulatory subunits, namely, cyclins, represent the major regulators of the cell cycle. By binding to the cyclins, CDKs control the transition of the cell cycle through the four sequential phases, G1, S, G2, and M phases. Their activity is tightly regulated by CDK inhibitors, such as p21(CIP1/WAF1) and phosphorylated retinoblastoma (pRb), to prevent abnormal proliferation [100,101,102]. Cancer cells are frequently characterized by deregulated CDK-cyclin complex activities, resulting in prolonged proliferation or inappropriate re-entry into the cell cycle [100]. It has been reported that natural and synthetic PPARγ ligands reduce cancer cell proliferation controlling the protein expression of several cell cycle regulators [103]. Additionally, 15d-PGJ2, rosiglitazone, and troglitazone decrease the gene expression of cyclin D1 inducing G1 cell cycle arrest [71,104]. Particularly, 15d-PGJ2 represses the cyclin D1 gene transcription reducing the binding of p300 to c-Fos for the cyclin D1 promoter activity, without affecting the expression of the CDK4 [71]. In contrast, troglitazone regulates the expression of cyclin D1 as well as the expression and the activity of CDK4 and CDK2, attenuating the Rb hyperphosphorylation, which is required for the G1-S phase cell cycle transition [104]. Rosiglitazone also promotes G0-G1 cell cycle arrest in breast cancer cells upregulating p53 protein expression and its effector p21 in a PPARγ-dependent manner [91]. Interestingly, the antiproliferative effects of PPARγ ligands are stronger in TNBC cells compared to ERα-positive breast cancer cells [105], addressing that PPARγ agonists could be a good therapeutic tool for the management of the more aggressive breast cancer subtypes. However, it has been demonstrated that PPARγ ligands can improve the efficacy of antitumoral drugs in ERα-positive breast cancer cells. Troglitazone has been demonstrated to exert a synergistic effect with tamoxifen in inducing growth inhibition, cell cycle arrest, and apoptosis in ERα-positive MCF-7 breast cancer cells. Indeed, the combined treatment of troglitazone (25 μM) with different doses of the selective estrogen receptor modulator tamoxifen (0–5 μM) induces an increase in the percentage of cells in cell cycle arrest at G1-S phases and a stronger downregulation of cyclin D1 expression than in cells treated with tamoxifen alone [105]. However, the clinical value of these results is still under investigation. Some of the molecular mechanisms by which PPARγ ligands regulate cell growth and cell cycle are depicted in Figure 2.

#### 5.1.2. Regulation of Cell Death

Cancer cells residing in a transient (quiescence) or permanently (senescence) cell cycle arrested state represent a double-edged sword for tumor progression since both quiescent and senescent cells might promote cancer stemness and chemoresistance [106]. Interestingly, besides inducing cell cycle arrest, PPARγ ligands can promote breast cancer cell death through apoptosis or autophagy.

##### Apoptosis

Apoptosis is a programmed cell death that occurs through two different pathways, namely, extrinsic and intrinsic pathways. Each pathway is initiated by the cleavage of caspases and carries on with a cascade of molecular events that determine the formation and degradation of apoptotic bodies [107,108]. Although the molecular mechanisms by which PPARγ ligands promote apoptosis are not fully understood, it has been reported that PPARγ agonists can trigger the two main signaling pathways of programmed cell death (Figure 2). In addition, 15d-PGJ2 induces apoptosis through mitochondrial dysfunction and reactive oxygen species (ROS) production, resulting in the activation of the extrinsic pathways [109]. Moreover, 15d-PGJ2 can also activate the intrinsic pathway, stimulating the release of the cytochrome c from the mitochondria into the cytoplasm and the cleavage of caspase 3,7 and poly (ADP-ribose) polymerase (PARP) [109]. However, the activation of the intrinsic pathway is not essential for the effects of 15dPG-J2 on breast cancer cell apoptosis since the treatment with the caspase inhibitor N-benzoylcarbanyl-Val-Ala-Asp-fluoro methylketone (zVAD-fmk) or the overexpression of B-cell lymphoma-2 (Bcl-2) in MCF-7 cells did not abrogate the 15d-PGJ2-induced cell death [109]. Moreover, the involvement of PPARγ in mediating these apoptotic effects has not been fully clarified. Recently, it has been reported that the biotinylated form of 15d-PGJ2 showed a stronger effect in inducing cell growth inhibition and apoptosis compared to 15d-PGJ2 [110]. Natural PPARγ ligands, including the ω-3 PUFAs and their conjugates, revealed proapoptotic effects through the activation of PPARγ [22,67,111]. It has been demonstrated that DHA induces apoptosis through the up-regulation of the tumor suppressor molecule syndecan-1 (SDC-1) in a PPARγ-dependent manner in MCF-7 cells [22]. Moreover, Rovito and coworkers have demonstrated that long-term treatment with the conjugates of EPA and DHA with dopamine (DA), EPADA, and DHADA, respectively, induced apoptosis in different human breast cancer cell lines, including MCF-7 (ERα positive), SKBR3 (ER/PR double negative and HER2 overexpressing), and MDA-MB-231 (triple negative) cells after the blockade of the autophagic flux. Indeed, treatment with EPADA and DHADA for 48 h increases the expression of p62, which expression levels inversely correlate with autophagic activity, and enhances the cleavage of both beclin-1 and caspase 9, suggesting the inhibition of the autophagy and the induction of intrinsic apoptosis [67]. Interestingly, it has been demonstrated that the proapoptotic effects of PPARγ ligands in breast cancer cells can be improved though multidrug approaches. Hydralazine, a drug used in the treatment of cardiovascular disease, has been found to boost the antiproliferative and proapoptotic effects of rosiglitazone in MDA-MB-231 cells. Although the molecular mechanism responsible for this effect is not fully understood, Jiang and coworkers suggested that hydralazine might reduce the hypermethylation of PPARγ preventing its inactivation [112]. Ciglitazone has been demonstrated to exert a synergic effect with a selective cyclooxygenase-2 (COX-2) inhibitor in inducing growth inhibition and apoptosis [113]. In addition, the combination of troglitazone with different RXR agonists has been demonstrated to induce growth inhibition in different cell lines and apoptosis in breast cancer cells [114]. Moreover, our research group has reported that low concentrations of rosiglitazone and the RXR ligand 9-cis-retinoic acid (9RA) induce the intrinsic apoptotic pathway in breast cancer cells through both dependent or independent p53 transcriptional activity [44,45]. Indeed, the combination of these drugs transactivates the tumor suppressor p53 promoter gene and enhances p53 protein expression and its target gene p21, increasing the release of the cytochrome c from the mitochondria into the cytoplasm and the cleavage of caspase-9 [44]. Interestingly, RXR and PPARγ agonists can also activate the intrinsic apoptosis through the upregulation of the proapoptotic Bid and the formation of a p53-Bid complex at the mitochondria promoting apoptosis [45]. Finally, it has been also demonstrated that PPARγ activated by rosiglitazone triggers extrinsic apoptotic process via a direct involvement of FAS/FAS ligand (FASL) signaling pathway. This occurs by PPARγ binding to the Sp1 sequence located within the FASL gene promoter leading to an upregulation of FASL expression, which causes caspase 8 cleavage and apoptotic cell death [115].

##### Autophagy

**Figure 3:**
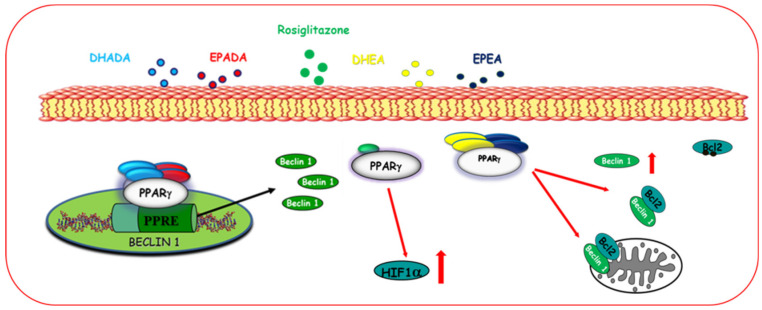
Activation of PPARγ induces autophagy in breast cancer cells. Natural and synthetic PPARγ increase hypoxia-inducible factor 1 (HIF1α) and Beclin-1 expression, which also results from the dissociation of the Beclin-1/Bcl2 complex thereby inducing autophagy.

Autophagy is a highly preserved catabolic process consisting of the degradation of intracellular components in order to maintain cell homeostasis [116]. Cellular stresses, such as starvation, stimulate the activation of the autophagic flux, which leads to the formation of the autophagosome and the degradation of its cargo after fusion with the lysosomes [117]. It has been demonstrated that PPARγ ligands trigger autophagy, which can precede and facilitate the activation of apoptotic cell death in breast cancer cells (Figure 3).

Zhou and coworkers found that troglitazone and rosiglitazone stimulate autophagy in triple-negative MDA-MB-231 breast cancer cells in a PPARγ-dependent manner through the upregulation of the hypoxia-inducible factor 1 (HIF1α), which is required for the hypoxia-induced autophagy [88]. Moreover, Rovito and coworkers have described that different conjugates of ω-3 PUFAs induce autophagy in breast cancer cells through PPARγ activation [21]. In particular, the conjugates of EPA and DHA with ethanolamine, EPEA and DHEA, respectively, promote the dissociation of the Beclin-1/Bcl2 complex, thus increase Beclin-1, which is crucial to induce autophagy. Moreover, EPEA and DHEA treatment reduces the phosphorylation of p38 and enhances the microtubule-associated protein light chain 3 (LC-3) levels, a specific membrane marker for the detection of early autophagosome formation [21]. Other two ω-3 PUFA derivatives, EPADA and DHADA, are able to induce autophagy as the first early event of PPARγ-dependent cell death. This event occurs by upregulating Beclin-1 transcriptional activity, which involves either PPARγ and RXR activation [67].

#### 5.1.3. Regulation of Motility and Invasion

**Figure 4:**
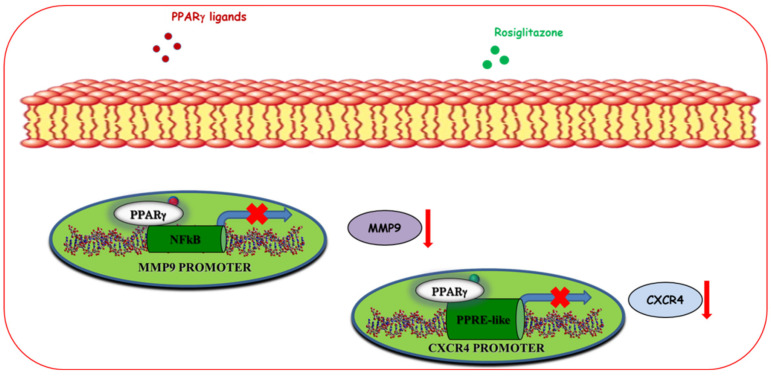
Ligand-activated PPARγ modulates motility and invasion of breast cancer cells. PPARγ agonists, through downregulation at transcriptional level of matrix metalloproteinases-9 (MMP-9) and C-X-C chemokine receptor type 4 (CXCR4), reduce both protein expression and inhibit breast cancer motility and invasion.

Breast cancer mortality mainly occurs because of the ability of cancer cells to metastasize to other sites. Breast cancer cell migration and invasion from the origin site to other second organs, including bone, lung, liver, and brain, represent the first steps of metastasis. PPARγ ligands have been found to counteract cell migration and invasion regulating the secretion of soluble factors and the chemokine networks in the tumor microenvironment (Figure 4).

In particular, it has been reported that non-toxic doses of 15d-PGJ2, DHA, and troglitazone reduce breast cancer cell invasion through the downregulation of the matrix metalloproteinases-9 (MMP9), a protease implicated in the extracellular matrix degradation in MCF-7 breast cancer cells [118,119,120]. The molecular mechanism by which PPARγ activated by ligands inhibits MMP9 expression occurs through suppression of 12-O-Tetradecanoylphorbol-13-acetate (TPA)-induced NF-κB sequence within MMP9 promoter [118]. Moreover, it has been described that rosiglitazone can block migration and invasion in breast cancer cells through the downregulation of CXCR4, one of the most important receptors involved in these processes [39]. Particularly, PPARγ directly binds to a newly identified PPRE-like sequence present in the CXCR4 promoter gene [39]. Interestingly, rosiglitazone also decreases the expression of CXCR4 in CAFs, which represent the principal source of stromal cell-derived factor 1 (SDF-1α) production, inhibiting their migratory capabilities and interfering with the autocrine and paracrine signaling loop acting to sustain breast tumor progression [39].

#### 5.1.4. Cross-Talk of PPARγ with Other Signal Transduction Pathways

**Figure 5:**
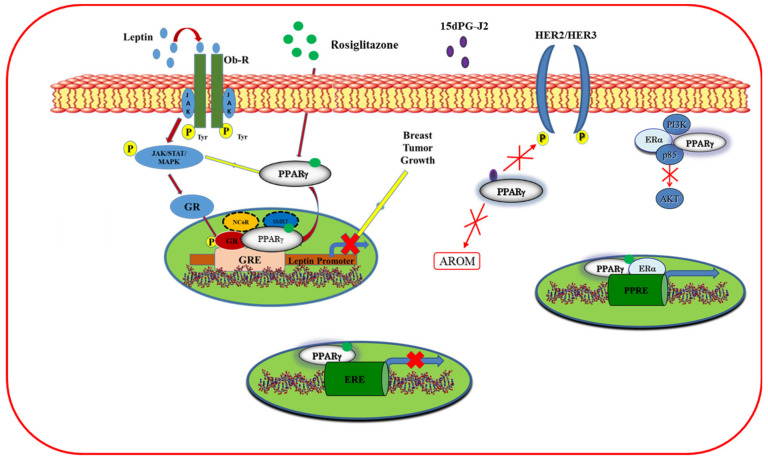
Cross-talk of PPARγ with other receptors in breast cancer cells. PPARγ ligands inhibit the leptin transcription and leptin-induced activation of JAK-STAT (Janus kinase/signal transducers and activators of transcription) and MAPK (mitogen-activated protein kinase) signaling. Moreover, ligand-activated PPARγ interferes with the human epidermal growth factor receptor 2/3 (HER2/HER3) pathway, decreases aromatase expression, trans represses the estrogen receptor α (ERα) target genes, and interacts with ERα antagonizing the activation of survival phosphatidylinositol 3-kinase (PI3K)/AKT (protein kinase B) signaling cascade, thus regulating breast cancer proliferation.

PPARγ agonists, other than their direct action through own receptor, can interfere with other signaling pathways, including ERα, HER2/HER3, and leptin (Figure 5).

It has been reported that a dual regulation occurs between ERα and PPARγ pathways. Indeed, ligand-activated PPARγ can bind to estrogen-responsive element (ERE) blocking the transcription of the ERα target genes [121], whereas ERα negatively interferes with PPRE-mediated transcriptional activity [122]. It has also been found that ERα binds to PPRE sequence sharing with PPARγ, the capability to bind AGGTCA half site contained in PPRE and ERE sequences [123]. Moreover, ERα physically interacts with PPARγ in a multiprotein complex involving p85, the regulatory subunit of the phosphatidylinositol 3-kinase (PI3K) survival pathway, even though ligand-activated PPARγ, exerting an opposite effect on the PI3K/AKT transduction pathway compared to ERα, induces breast cancer cell growth inhibition. Interestingly, the antiproliferative effects exerted by PPARγ ligands can be potentiated in the presence of specific ERα antagonists, supporting the potential role of PPARγ ligands in multidrug regimens for the treatment of hormone-positive breast cancer [123]. It has been reported that PPARγ ligands can also modulate the estrogen synthesis. In particular, troglitazone and 15d-PGJ2 were able to reduce the aromatase expression in breast adipose stromal cells treated with oncostatin M or tumor necrosis factor (TNF)α and dexamethasone in a concentration-dependent manner, thus blocking the conversion of androgens to estrogens [124]. Moreover, Pignatelli and coworkers have described a cross-talk between PPARγ and the membrane tyrosine kinase receptor HER2/Erb signal, which is involved in breast cancer tumor progression. Indeed, 15d-PGJ2 inhibited the neuregulin-induced phosphorylation of HER2 and HER3, triggering apoptosis, cell growth inhibition, and differentiation [125]. In an in vitro and in vivo experimental approach, rosiglitazone antagonizes the proliferative effects exerted by leptin in breast cancer cells and abrogates the leptin-induced tumor growth in nude mice implanted with MCF-7 cells [126]. Specifically, the molecular mechanism by which rosiglitazone counteracts leptin action is related to the repression of leptin transcription via the recruitment of two PPARγ corepressors, N-CoR and SMRT, to the glucocorticoid-responsive element in the promoter region of the leptin. In addition, rosiglitazone reduces the expression of the leptin receptors, antagonizes the leptin signal, and inhibits the MAPK/STAT3/Akt pathway [126], suggesting that PPARγ agonists may have a potential role in the treatment of breast cancer patients.

### 5.2. In Vivo Studies

Although the importance of PPARγ expression in modulating in vivo breast tumor formation is still controversial [127], the effects of several PPARγ ligands in controlling breast carcinogenesis have been confirmed in animal models. Troglitazone alone or in combination with the RXR ligand all-trans-retinoic acid (ATRA) has been demonstrated to reduce the tumor size and weight in triple-immunodeficient mice inoculated with MCF-7 breast cancer cells, inducing tumor growth inhibition in vivo. Interestingly, troglitazone caused apoptotic effects in the tumors, without inducing toxic effects in the mice. However, more marked apoptotic changes were observed in mice treated with the combination of troglitazone and ATRA, confirming the ability of PPARγ agonists to exert synergistic effects with other compounds [128]. Moreover, MDA-MB-231 cells treated with 15d-PGJ2 prior to inoculation in BALB/C nude mice have demonstrated a decreased capability to form tumors compared to untreated control [129]. Rosiglitazone was also able to prevent the leptin-induced tumor growth in nude mice after 12 weeks of treatment [126]. Notably, the administration of rosiglitazone was well tolerated because no significant differences in the mean weights or in the histologic features of the major organs (liver, lung, spleen, and kidney) were observed between vehicle-treated mice and those that received treatment [126]. Moreover, a novel synthetic PPARγ ligand, the triterpenoid 2-Cyano-3,12-dioxooleana-1,9-dien-28-oic acid (CDDO), was found to lower the tumor growth of MDA-MB-435 breast cancer cells implanted in nude mice [130]. Suppression of tumor cell growth by ω-3 PUFAs has been confirmed in vivo using cancer animal models mainly represented by xenograft nude mice implanted with different tumor cell types [131,132,133,134,135] and by transgenic rodent models [136,137,138]. Indeed, dietary supplementation with ω-3 PUFAs has been reported to increase PPARγ protein expression, which was concomitant with a reduction of tumor burden in rats with induced mammary carcinogenesis [139]. All the above considerations support the opportunity of reaching therapeutic levels of these compounds in experimental in vivo models by dietary means as well as by the pharmacological use of receptor agonists to achieve potential anticancer benefits.

### 5.3. Clinical Studies

Despite the large number of preclinical studies investigating the role of ligand-activated PPARγ in breast cancer, only a few clinical trials have been conducted, and the results mainly address the potential of PPARγ agonists on breast cancer risk in healthy women and on the modulation of chemotherapy-related side effects in breast cancer patients. A phase II study of troglitazone was carried out in patients with advanced breast cancer refractory to hormonal and chemotherapy agents, but it demonstrated only a few clinical benefits. Indeed, only 3 of 21 enrolled patients showed a stable disease at 8 weeks, whereas no objective tumor responses or stabilization for 6 months were observed [19]. In contrast, a pilot trial of rosiglitazone in early-stage breast cancer patients revealed that the administration of rosiglitazone between the time of diagnostic biopsy and definitive surgery may be relevant for breast cancer treatment. Although rosiglitazone was not found to reduce breast tumor proliferation, it increased the serum adiponectin levels and reduced insulin resistance, which is correlated with breast cancer risk [140]. Till date, another clinical trial using synthetic PPARγ agonist rosiglitazone has been reported in the ClinicalTrials.gov databases, whereas 40 clinical studies have been conducted with dietary ω-3 PUFAs in breast cancer. Interestingly, 17 clinical trials have been completed and mainly assessed the role of EPA and DHA in reducing breast cancer risk and controlling the side effects of chemotherapy thus impacting breast cancer survivors. Details of these studies are included in Table 3.

## 6. Conclusions

Although substantial advances have been made in breast cancer diagnosis and treatment over the last years, this disease remains a major health problem among women worldwide. Despite limited in perspective, the overall suggestions from accumulated data relating to PPARγ and breast cancer strongly highlight that this receptor may have an important role as tumor suppressor. Hence, numerous preclinical studies indicate the ability of natural as well as synthetic PPARγ ligands to modulate the tumor cell signaling mechanisms and/or the cross-talk with various extracellular factors and intracellular pathways controlling the growth, survival, and invasive/metastatic behavior of breast cancer cells. Evidence also support that PPARγ activation results in inhibition of breast tumor growth either in vivo models or in vitro breast cancer cells. Despite these promising preclinical results, the published clinical studies on synthetic PPARγ ligands in breast cancer patients are limited and generally disappointing for their potential side effects. In this scenario, understanding the mechanism of action of natural compounds, such as ω-3 PUFAs, could be useful for the development of new pharmacological tools in breast cancer. Moreover, one of the future priorities for a rational use of PPARγ agonists will be the identification of the subgroups of breast tumors currently lacking effective therapeutic options, which may really benefit from novel adjuvant therapeutic interventions. Finally, further investigation and additional clinical trials will confirm whether PPARγ agonists may represent a valuable tool in the prevention and treatment of breast cancer.

## Figures and Tables

**Table 1:** Class, common as well as IUPAC name, and chemical structure of natural peroxisome proliferator-activated receptor gamma (PPARγ) ligands.

Class	Common Name	IUPAC NAME	Chemical Structure	References
ω-3 PUFAs ^1^	Eicosapentaenoic acid (EPA) 20:5(ω-3)	(5*Z*,8*Z*,11*Z*,14*Z*,17)-Eicosa-5,8,11,14,17-pentaenoic acid	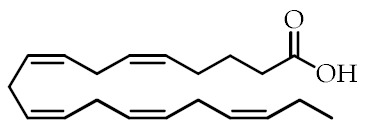	[64,65]
	Docosahexaenoic acid (DHA) 22:6(ω-3)	(4*Z*,7*Z*,10*Z*,13*Z*,16*Z*,19*Z*)-Docosa-4,7,10,13,16,19-hexaenoic acid	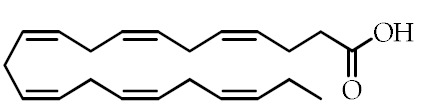	[57,66]
ω-3 PUFA conjugates	Eicosapentaenoyl ethanolamine (EPEA)	(5*Z*,8*Z*,11*Z*,14*Z*,17)-*N*-(2-Hydroxyethyl)-eicosa-5,8,11,14,17-pentaenamide	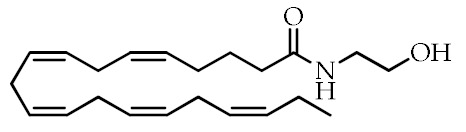	[21]
	Docosahexaenoyl ethanolamine (DHEA)	(4*Z*,7*Z*,10*Z*,13*Z*,16*Z*,19*Z*)-*N*-(2-Hydroxyethyl)-docosa-4,7,10,13,16,19-hexaenamide	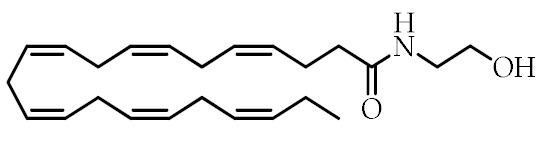	[21]
	Eicosapentaenoyl dopamine (EPADA)	(5*Z*,8*Z*,11*Z*,14*Z*,17)-*N*-(3,4-Dihydroxyphenethyl)eicosa-5,8,11,14,17-pentaenamide	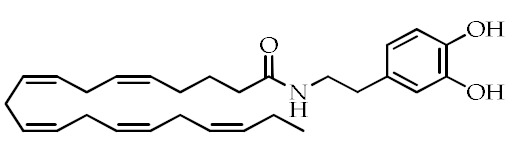	[67]
	Docosahexaenoyl dopamine (DHADA)	(4*Z*,7*Z*,10*Z*,13*Z*,16*Z*,19*Z*)-*N*-(3,4-Dihydroxyphenethyl)docosa-4,7,10,13,16,19-hexaenamide	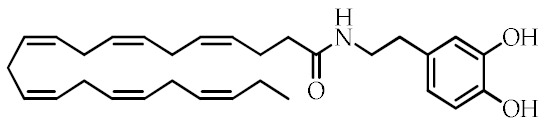	[67]
	Docosahexaenoyl serotonin (DHA-5-HT)	(4*Z*,7*Z*,10*Z*,13*Z*,16*Z*,19*Z*)-*N*-[2-(5-hydroxy-1*H*-indol-3-yl)ethyl]-docosa-4,7,10*,*13,16,19-hexaenamide	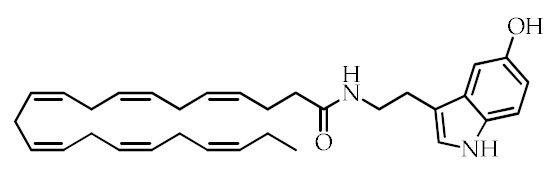	[30]
ω-6 PUFAs	Linoleic acid (LA) 18:2(ω-6)	(9*Z*,12*Z*)-Octadeca-9,12-dienoic acid	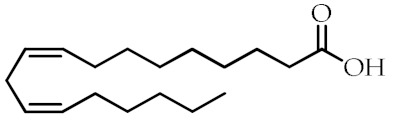	[68]
	9-Hydroxyoctadecadienoic acid(9-HODE)	(9*S*,10*E*,12*E*)-9-Hydroxyoctadeca-10,12-dienoic acid	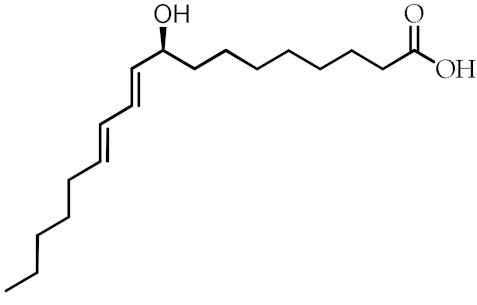	[69,70]
	13-Hydroxyoctadecadienoic acid (13-HODE)	(9*Z*,11*E*,13*S*)-13-Hydroxyoctadeca-9,11-dienoic acid	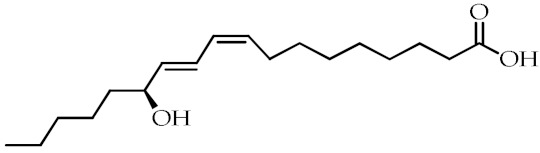	[69,70]
	15-Deoxy-Δ-^12,14^-prostaglandin J2 (15d-PGJ2)	(*Z*)-7-((*S*,*E*)-5-((*E*)-oct-2-en-1-ylidene)-4-oxocyclopent-2-en-1-yl)hept-5-enoic acid	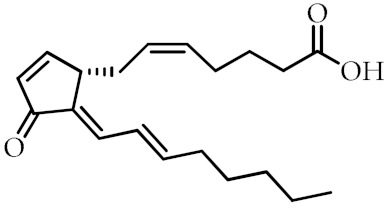	[71]
Conjugated linoleic acid (CLA)	(9*Z*,11*E*)-CLA	(9*Z*,11*E*)-Octadeca-9,11-dienoic acid	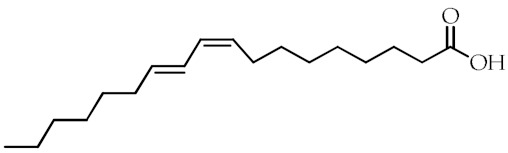	[72]
	(9*E*,11*E*)-CLA	(9*E*,11*E*)-Octadeca-9,11-dienoic acid	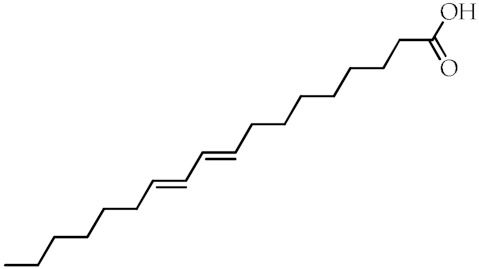	[72]
	(9*Z*,11*Z*)-CLA	(9*Z*,11*Z*)-Octadeca-9,11-dienoic acid	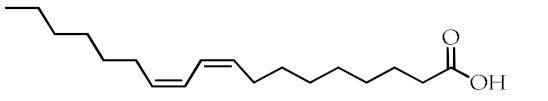	[72]
	(10*E*,12*E*)-CLA	(10*E*,12*E*)-Octadeca-9,11-dienoic acid	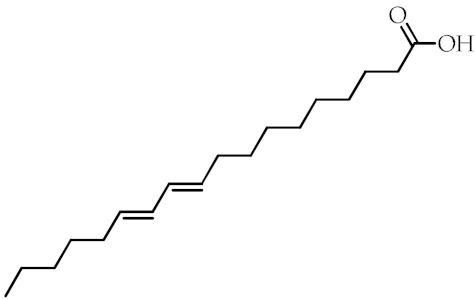	[72]
Nitrated fatty acids	Nitrolinolenic acid	(9*Z*,12*Z*)-2-Nitrooctadeca-9,12-dienoic acid	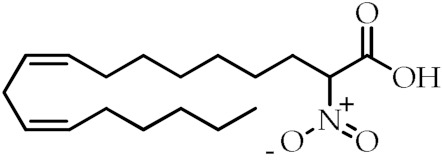	[60]
	10-Nitrooleic acid	(9*Z*)-10-Nitrooctadec-9-enoic acid	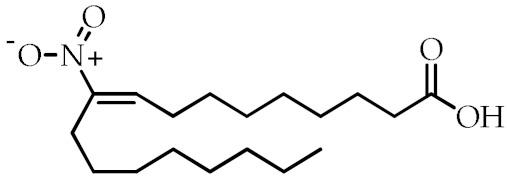	[61]
Bioactive compounds	Genistein	5,7-Dihydroxy-3-(4-hydroxyphenyl)-4*H*-chromen-4-one	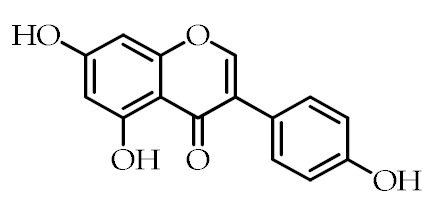	[73]
	Quercetin	2-(3,4-Dihydroxyphenyl)-3,5,7-trihydroxy-4*H*-chromen-4-one	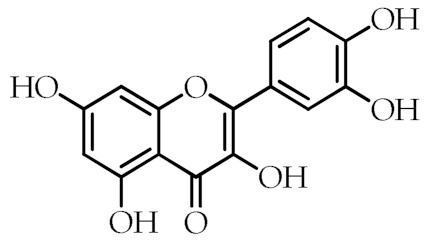	[74]
	Luteolin	2-(3,4-Dihydroxyphenyl)-5,7-dihydroxy-4*H*-chromen-4-one	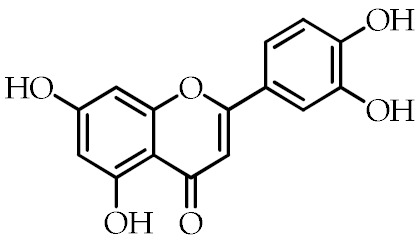	[75]

^1^ PUFAs: polyunsaturated fatty acids.

**Table 2:** Class, common as well as IUPAC name, and chemical structure of synthetic PPARγ ligands.

Class	Common Name	IUPAC Name	Chemical Structure	References
TDZs ^1^	Troglitazone	5-[[4-[(6-Hydroxy-2,5,7,8-tetramethyl-3,4-dihydrochromen-2-yl)methoxy]phenyl]methyl]-1,3-thiazolidine-2,4-dione	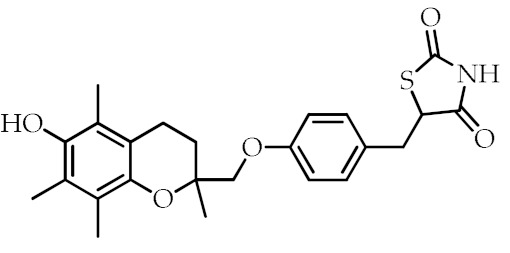	[88]
	Ciglitazone	5-{4-[(1-Methylcyclohexyl)methoxy]benzyl}-1,3-thiazolidine-2,4-dione	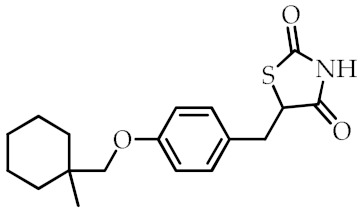	[89]
	Pioglitazone	5-[[4-[2-(5-Ethylpyridin-2-yl)ethoxy]phenyl]methyl]-1,3-thiazolidine-2,4-dione	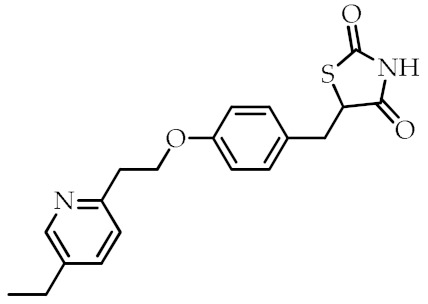	[90]
	Rosiglitazone	5-(4-(2-(Methyl(pyridin-2-yl)amino)ethoxy)benzyl)thiazolidine-2,4-dione	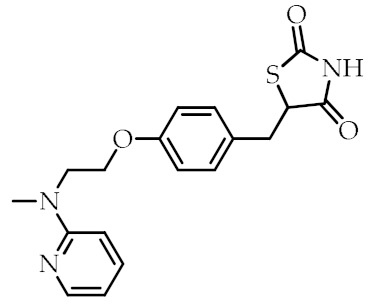	[91]
	Edaglitazone	5-[[4-[2-(5-Methyl-2-phenyl-1,3-oxazol-4-yl)ethoxy]-1-benzothiophen-7-yl]methyl]-1,3-thiazolidine-2,4-dione	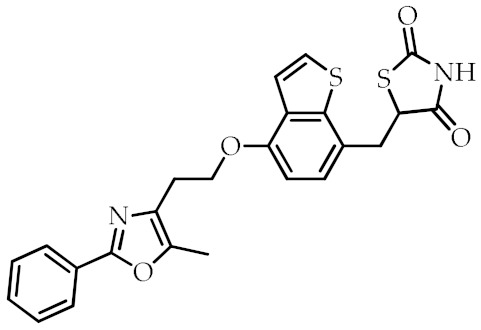	[78]
	Efatutazone	5-[(4-{[6-(4-Amino-3,5-dimethylphenoxy)-1-methyl-1*H*-1,3-benzodiazol-2-yl]methoxy}phenyl)methyl]-1,3-thiazolidine-2,4-dione	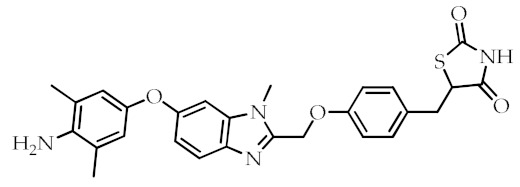	[80]
	Rivoglitazone	5-({4-[(6-Methoxy-1-methyl-1*H*-benzimidazol-2-Yl)methoxy]phenyl}methyl)-1,3-thiazolidine-2,4-dione	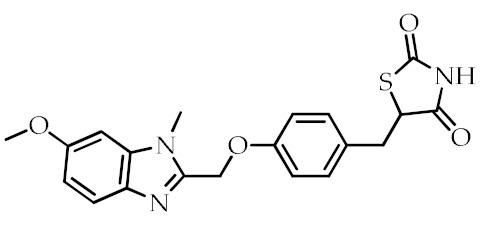	[79]
SPPARMs ^2^	GW0072	3-(4-(4-Carboxyphenyl)butyl)-2-heptyl-4-oxo-5-thiazolidine	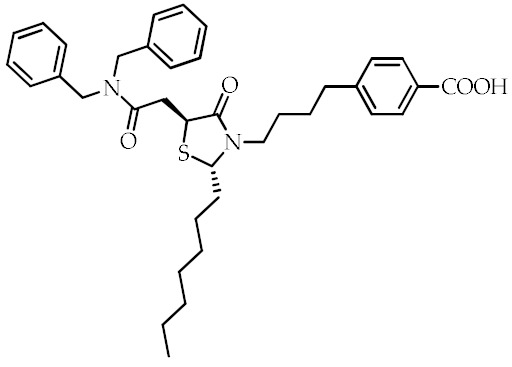	[92]
	Fmoc-l-leucine	2-(9*H*-Fluoren-9-ylmethoxycarbonylamino)-4-methylpentanoic acid	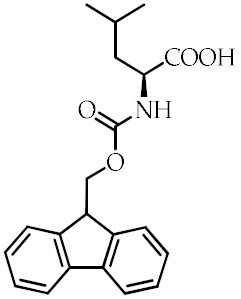	[93]
	Balaglitazone	5-(4-((3-Methyl-4-oxo-3,4-dihydroquinazolin-2-yl)methoxy)benzyl)thiazolidine-2,4-dione	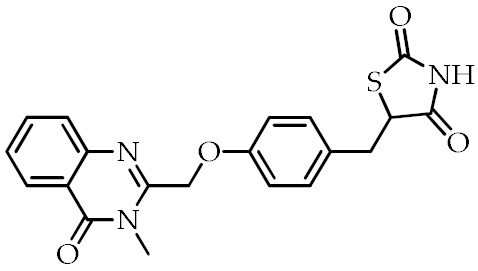	[94]
	Netoglitazone	5-[[6-[(2-Fluorophenyl)methoxy]naphthalen-2-yl]methyl]-1,3-thiazolidine-2,4-dione	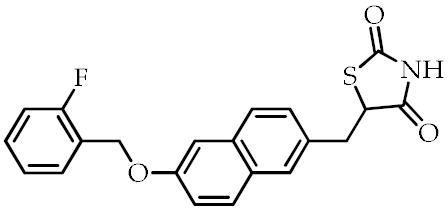	[95]
NSAIDs ^3^	Sulindac sulfide	5-Fluoro-2-methyl-1-[*p*-(methylthio)benzylidene]indene-3-acetic acid	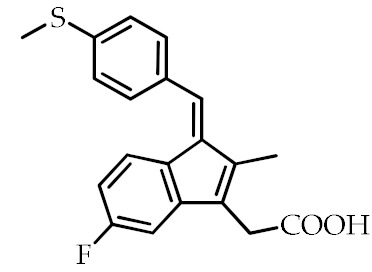	[87]
	Diclofenac	2-[2-(2,6-Dichloroanilino)phenyl]acetic acid	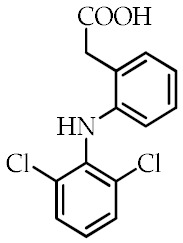	[87]
	Indomethacin	2-[1-(4-Chlorobenzoyl)-5-methoxy-2-methylindol-3-yl]acetic acid	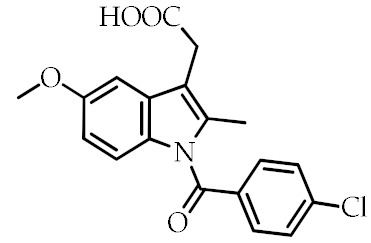	[68,84,87]
	Ibuprofen	2-(4-Isobutylphenyl)propanoic acid	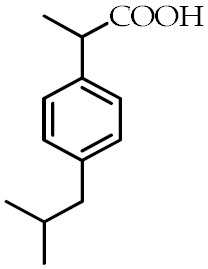	[87]

^1^ TDZs: thiazolidinediones; ^2^ SPPARMs: selective PPARγ modulators; ^3^ NSAIDs: nonsteroidal anti-inflammatory drugs.

**Table 3:** List of the studies on PPARγ agonists and breast cancer at www.clinicaltrials.gov.

NCT	Intervention/Treatment	Phase	Eligibility Criteria, Primary Outcome and Purpose	References
NCT01282580	-Lovaza -ω-3 fatty acid capsules -Dietary fish (canned salmon, albacore)	I	**Eligibility criteria**: female (≥18 years) having increased risk for breast cancer based on family and personal history with a normal mammogram in the past 12 years and >1 years from pregnancy, lactation, or chemotherapy. **Primary outcome**: fatty acid profiles of breast adipose tissue. **Purpose**: to determine the effects of increased fish consumption on serum and breast fat tissue fatty acids and to assess adherence and tolerability of increased dietary intake of fish relative to an ω-3 fatty acid supplement.	[141]
NCT02150525	-ω-3 fatty acids —Placebo	II	**Eligibility criteria**: postmenopausal women (45–65 years) with a history of breast cancer after 12 months from surgery and 3 months from completion of chemotherapy. Subjects must present at least one symptom of atrophic vaginitis. **Primary outcome**: improvement of vaginitis’ symptoms by oral ω-3 fatty acids. **Purpose**: to study the effectiveness of ω-3 fatty acid on atrophic vaginitis in postmenopausal breast cancer patients.	NA
NCT02101970	-Weight Loss + ω-3 fatty acids -Weight Loss + Placebo	NA	**Eligibility criteria**: female having evidence of hyperplasia with Masood score of 13 or higher, 500 or more epithelial cells on cytology slide of screening random periareolar fine-needle aspiration, and reasonable hematopoietic, kidney, and liver function. **Primary outcome**: dropout rate. **Purpose**: to determine if high dose supplementation with the ω-3 fatty acids EPA and DHA, when added to a weight loss program, is well tolerated and if there is an increase in the favorable change in blood and tissue breast cancer risk factors when compared to weight loss alone.	NA
NCT02816125	-Diet supplemented with EPA + DHA -Dietary fat at less than 20% energy + EPA + DHA	III	**Eligibility criteria**: menstruating and premenopausal women (≥18 years). **Primary outcome**: fatty acid incorporation into red blood cells and in cell material from nipple aspirate fluid. **Purpose**: to examine the effect of the combined treatment with EPA and DHA on breast cancer risk factors in healthy premenopausal women.	[142]
NCT00723398	-Raloxifene -Lovaza -Lovaza + Raloxifene	NA	**Eligibility criteria**: no smoker postmenopausal women (35–70 years) without hormone-replaced therapy for at least 6 months. **Primary outcome**: absolute breast density. **Purpose**: to evaluate the effects of Raloxifene alone or in combination with omega-3 fatty acids on breast cancer development markers in postmenopausal women.	[143]
NCT01823991	-Lovaza -VitaBlue -Placebo	Early I	**Eligibility criteria**: women (40–70 years) having stage II-IIIA of breast cancer after completion of adjuvant treatment with chemo- and/or radiotherapy in the past 6 months. **Primary outcome**: cognitive function scores with intervention. **Purpose**: to evaluate the safety of nutritional intervention with n-3 fatty acids and blueberry anthocyanins on cognitive performance.	NA
NCT01869764	-ω-3 fatty acids -Placebo	II	**Eligibility criteria**: newly diagnosed women (≥18 years) having in situ carcinoma and stage I to III of breast cancer that will receive breast surgery at least after 7 days from the day of enrollment. Tumor size of at least 1 cm. **Primary outcome**: ω-3 fatty acid levels in breast tissues in plasma and in erythrocytes before and after surgery. **Purpose**: to study ω-3 fatty acid effects in patients with breast cancer.	NA
NCT02352779	-Low doses of ω-3 fatty acid -High dose of ω-3 fatty acid -Placebo	NA	**Eligibility criteria**: women having a confirmed diagnosis of breast cancer and undergone some type or combination of standard adjuvant treatment. Patients must have cancer-related fatigue. **Primary outcome**: mean change and standard deviation in cancer-related fatigue. **Purpose**: to study ω-3 fatty acid in reducing cancer-related fatigue.	[144]
NCT01385137	-ω-3-fatty acids -Placebo	III	**Eligibility criteria**: postmenopausal women with estrogen-receptor positive and/or progesterone-receptor positive invasive breast adenocarcinoma (I-IIIA stage) currently taking a third-generation aromatase inhibitor; patients must be subjected to modified radical mastectomy or breast-sparing surgery. **Primary outcome**: Brief Pain Inventory (BPI) Worst Pain/Stiffness Score. **Purpose**: to study ω-3 fatty acid effects in muscle, bone pain, and stiffness in breast cancer patients receiving hormone therapy.	[145]
NCT01252277	-Lovaza	II	**Eligibility criteria**: premenopausal female (18–54 years) having a breast cancer risk evaluated on the basis of several criteria. **Primary outcome**: proportion of subjects that complete an intervention. **Purpose**: to evaluate the effects of Lovaza on breast cancer biomarkers in premenopausal patients.	[146]
NCT01252290	-Lovaza	II	**Eligibility criteria**: postmenopausal women (25–69 years) having a breast cancer risk evaluated on the basis of several criteria. **Primary outcome**: proportion of subjects that complete the intervention. **Purpose**: to evaluate the effects of Lovaza on breast cancer biomarkers in postmenopausal patients.	[147]
NCT00627276	-ω-3 fatty acids-Placebo	NA	**Eligibility criteria**: breast cancer patients having confirmed diagnosis of ductal carcinoma in situ and/or atypical ductal hyperplasia. **Primary outcome**: genetic evaluation of markers for breast cancer risk and progression, fatty acids profile, occurrence of ductal carcinoma in situ and/or atypical ductal hyperplasia or invasive cancer in tissue samples. **Purpose**: to study the effects of ω-3 fatty acids in patients with ductal carcinoma in situ and/or atypical ductal hyperplasia.	NA
NCT01824498	-Low-fat diet -Low fat with high ω -3 diet-High-fat diet	NA	**Eligibility criteria**: postmenopausal women (45–70 years) having BMI between 19 and 29. Consumption of a “typical” American diet. **Primary outcome**: plasma sex hormone levels, Estradiol. **Purpose**: to determine whether diets designed to increase plasma ω-3 concentrations will favorably affect sex hormone distribution in women in a direction associated with reduced risk of sex hormone-mediated cancer development.	[148,149,150,151]
NCT02062255	-Aspirin -ω-3 EPA and DHA -Aspirin + ω-3 EPA and DHA	Early phase I	**Eligibility criteria**: postmenopausal women (≥18 years). **Primary outcome**: prostaglandin E2, aromatase, pro-inflammatory cytokines, steroids, and lipids. **Purpose**: to study the impact of COX2 on sera biomarkers from obese subjects.	NA
NCT01821833	-ω-3 fatty acids + paclitaxel -Placebo + paclitaxel	NA	**Eligibility criteria** women (≥18 years) with breast cancer or ovarian cancer who will receive paclitaxel treatment at least for 2 months. **Primary outcome**: mean severity of pain. **Purpose**: to study the effects of ω-3 fatty acids on pain of cancer patients.	NA
NCT01127867	-DHA	NA	**Eligibility criteria**: postmenopausal women (40–70 years) having low serum estradiol level (<40 ng/mL) and BMI 35–50. **Primary outcome**: evaluation of monocyte aggregations and macrophage markers in fat biopsies. **Purpose**: to study the effects of DHA in decreasing inflammation in fat tissue and reducing breast cancer risk.	[152]
NCT01902745	-Fatigue-reduction diet (typical caloric intake and replacement of some calories with the following foods: whole grains, vegetables, fruit, fatty fish, and nuts and/or seeds)	NA	**Eligibility criteria**: breast cancer patients (≥18 years) having completed the cancer-related treatments and showing persistent, moderate, or severe fatigue despite standard treatment. **Primary outcome**: fatigue in breast cancer survivors. **Purpose**: to expand the data on fatigue-reduction diet in breast cancer survivors	[153]
NCT00933309	-Exemestane -Exemestane + Avandamet (rosiglitazone and metformin)	I	**Eligibility criteria**: postmenopausal women with BMI ≥ 25 kg/m^2^ having estrogen receptor positive and/or progesterone receptor positive breast cancer along with clinical evidence of metastasis. **Primary outcome**: Dose-limiting toxicity (DLT). **Purpose**: to define the highest tolerable dose of Avandamet in combination with exemestane in postmenopausal obese breast cancer patients.	[154]
